# Does high-intensity work intensify the imbalance between health and income? Evidence from rural China

**DOI:** 10.3389/fpubh.2025.1589364

**Published:** 2025-06-02

**Authors:** Mao Zhao, Yu Xiong, MiaoJie Wang, YuHang Zhang, YaJing Cao

**Affiliations:** ^1^School of Management, Yunnan Normal University, Kunming, China; ^2^School of Economics, Yunnan Normal University, Kunming, China

**Keywords:** high-intensity work, reward for work, coupling coordination degree, endogenous switching probit model, Chinese farmers

## Abstract

Understanding the interplay between health and income among rural populations is essential for achieving sustainable development and reducing global inequalities. This paper evaluated the health-income coupling coordination degree (CCD) among farmers by utilizing data from the 2018 China Labor Force Dynamics Survey. It analyzed the impact of high-intensity work on the CCD among farmers by using the endogenous switching probit model (ESP). Additionally, this paper investigated the individual variations in the impact effect. This paper reveals that high-intensity work has a detrimental impact on the CCD among farmers. Farmers engaged in high-intensity work have an 8.527% higher probability of experiencing imbalanced CCD than those with low-intensity work. Furthermore, the adverse effect of high-intensity work on the CCD is more pronounced among farmers working in a different location than those working locally. These findings hold significance for assisting developing countries worldwide in achieving prosperity for farmers and rural development.

## 1 Introduction

In early 2021, China officially announced the achievement of its poverty eradication goal, eliminating absolute and regional poverty,^1^ and making significant contributions to global poverty reduction. Subsequently, rural revitalization has taken precedence as one of China’s primary focuses in the three rural areas (1). In 2022, Chinese leaders emphasized the comprehensive promotion of rural revitalization during the twentieth National Congress of the Communist Party of China (CPC). An essential objective of rural revitalization is to ensure sustainable growth in farmers’ income and continuously enhance their sense of well-being, contentment, and security.^2^ Thus, even after reaching the poverty eradication target, increasing farmers’ income remains a significant priority in China’s approach to the three rural areas (2). According to data from the China Rural Statistical Yearbook for 2021, Chinese farmers’ wage income and agricultural business income were recorded as RMB 7,958.1 and RMB 4,291.7, respectively. These figures represent a notable increase of 44.73 and 26.56% compared to 4 years ago, indicating significant and rapid growth. Additionally, Li (3) found that over 70% of farmers in China work more than the legally required 8 h a day, which suggests that a portion of the substantial income growth among Chinese farmers can be attributed to longer daily working hours, aligning with the adage “hard work makes you wealthy.” The reasons for this phenomenon can be attributed to several factors. Firstly, the migration of farmers to non-agricultural sectors and the aging of the rural population have led to a scarcity of young and robust rural labor. As Chinese agriculture largely relies on smallholder farming, which demands significant labor, farmers often face acute labor shortages during busy farming periods (4). They extend their daily working hours to meet the demands of agricultural production. Secondly, farmers’ productivity is limited by factors such as social capital and education level (5, 6). Farmers often seek additional income opportunities during their leisure time through side jobs (7). However, these jobs are often irregular and physically demanding, such as odd jobs and vending (8), resulting in lower income stability and reduced earnings. To bolster their income, farmers engaged in sideline work often extend their daily working hours (9).

Increased working hours result in higher work intensity (10), leading to substantial income gains. However, this intense work is also linked to health issues caused by “overwork” (11, 12). Health problems related to “overwork” can be exacerbated by lower occupational status, which increases farmers’ vulnerability to risk factors (13, 14) and worsens the adverse effects of the work environment on health (15). Currently, the health status of Chinese farmers is concerning, as over 60% of rural households have at least one sick individual (16), and chronic diseases are prevalent (17). Farmers with chronic illnesses often require long-term medication to manage their conditions, but limited knowledge and adherence to medicines can compromise the effectiveness of treatment (18), leading to complications and heightened health risks, including the possibility of returning to poverty. Approximately 40% of families in China experience “impoverishment due to illness” (19), with increasing health expenditures for chronic disease treatment becoming a significant factor pushing farmers back into poverty (20). Though the government introduced a new rural cooperative medical scheme to address the risk of illness-induced poverty among farmers, the complex reimbursement process and low reimbursement rate of rural medical insurance still result in high out-of-pocket expenses for farmers (16, 21, 22). Consequently, many farmers resort to “delaying minor illnesses and resisting major ones.” In light of this, the increased work intensity carries the risk of “poverty due to illness,” which contradicts the notion that “hard work makes you wealthy.”

Therefore, the critical question for Chinese farmers is understanding the precise impact of high-intensity work on their wealth. Does it create a win-win scenario where health and income receive equal attention, leading to a harmonious coexistence? Alternatively, does it result in a trade-off situation, akin to the idiom “trying to have one’s cake and eat it too,” leading to a dissonance between health and income? As previously mentioned, farmers’ health and income levels interact and influence each other, with the prosperity of farmers’ wealth dependent on achieving a balance and harmonious development between the two aspects. In physics, “coupling” describes the interaction between subsystems, reflecting how multiple subsystems influence each other through various interactions (23). So, this study refers to the dynamic relationship between farm households’ health and income levels, where they interact and constrain each other, as a “coupling.” Specifically, the study utilizes data from the China Labor Force Dynamic Survey (CLDS) published by the Social Survey Center of Sun Yat-sen University in 2018. The study calculates the degree of coupling coordination between these indices by constructing a composite index for farm households’ income and health levels. Furthermore, the endogenous switching probit model (ESP) is employed to analyze the impact of high-intensity work on farmers’ income while exploring the individual variability of this effect. The potential incremental contributions of this study can be observed in the following aspects: First, existing studies analyzing the relationship between work intensity, health, and income predominantly rely on propensity score matching (PSM). However, PSM only addresses selection bias from observable factors, failing to account for endogeneity caused by unobservable factors such as individual stress resilience or occupational preferences. This study employs the endogenous switching probit (ESP) model, which resolves self-selection bias through instrumental variables, offering a more robust methodological framework for causal inference in labor economics. Second, traditional research often treats health and income as independent variables, overlooking their dynamic interplay. This study introduces the coupling coordination model, a novel approach adapted from physics, to quantify the synergistic evolution between health and income. Unlike conventional regression analyses that examine health or income in isolation, this model breaks away from the unidirectional perspective, revealing a vicious cycle: high-intensity work - health deterioration - unsustainable income growth. The findings challenge the conventional “hard work leads to prosperity” theory in development economics, expanding its theoretical boundaries.

## 2 Theoretical analysis

### 2.1 The impact of high-intensity work on the CCD among farmers

High-intensity work will affect the CCD by influencing farmers’ physical and mental health. First, from a physiological perspective, Chinese farmers typically face demanding work conditions characterized by strenuous labor, poor working environments, and low-income levels (24, 25). High-intensity work makes it difficult for farmers to obtain quality rest and often leads to unhealthy habits such as lack of exercise, smoking, and drinking. These negative behaviors disrupt circadian rhythms and metabolic functions, weaken immunity, and increase the risk of chronic diseases (26). When illness occurs, farmers confront substantial medical expenses. Some households avoid treatment due to high costs, which leads to severe health deterioration and reduced work efficiency (27), ultimately lowering income. They may extend working hours to compensate, but this further strains their bodies, trapping them in a vicious cycle. Other households opt for active treatment, which improves health but significantly increases expenses, reducing income and still resulting in imbalance. To offset this income gap, they intensify their workload, which further impairs sleep, elevates disease risk, and drives up medical costs, trapping them in a vicious cycle.

Additionally, from a psychological standpoint, the stress and exhaustion from high-intensity work generate anxiety, depression, and other negative emotions. Prolonged psychological distress further deteriorates sleep quality, creating a bidirectional psychophysiological effect that exacerbates chronic disease risks. Mental burdens also diminish farmers’ work motivation and efficiency, making income growth even more challenging. Based on this, the following hypotheses are proposed for this study:

*H1*: High-intensity work can lead to a decline in the CCD among farmers, resulting in “losing sight of the other.”

### 2.2 Heterogeneous impacts of high-intensity work on the CCD among farmers

High-intensity work exerts more pronounced adverse effects on the CCD among migrant farmers, which stems from labor mobility segmentation. Geographic displacement triggers psychological adaptation stress, making migrant farmers particularly vulnerable to homesickness and related disorders (28), directly impairing their health capital accumulation. Labor market segmentation systematically disadvantages migrant workers in wage compensation (29), forcing them to compensate for income gaps through extended working hours. The household registration system creates welfare segmentation, restricting migrant workers’ access to local social security resources (30), with medical insurance portability barriers significantly amplifying health risk exposure. These institutional constraints collectively create a triple burden where migrant farmers work harder yet receive less health protection, ultimately degrading their CCD. Based on this, the following hypotheses are proposed for this study:

*H2a*: There is labor mobility heterogeneity in the effect of work intensity on the CCD, i.e., the negative impact of high-intensity work on the CCD is more pronounced for farmers who work off-site.

China’s uneven regional development has created significant labor market segmentation (9). In eastern regions, advanced industrial systems and robust social safety nets jointly mitigate health-income trade-offs: higher wage premiums partially offset health depreciation from intensive labor. At the same time, superior healthcare accessibility reduces income erosion from health risks. In non-eastern regions, however, market segmentation manifests differently. Agriculture-dependent economies constrain off-farm employment opportunities, while weak local fiscal capacity limits medical coverage. This dual constraint of “low income–low protection” traps farmers in a vicious cycle of health-income imbalance. Under comparable conditions, non-eastern farmers endure heavier workloads yet receive weaker health safeguards, resulting in poorer CCD. Based on this, the following hypotheses are proposed for this study:

*H2b*: There is labor market heterogeneity in the effect of work intensity on the CCD, i.e., the negative impact of high-intensity work on the CCD is more pronounced for farmers in non-Eastern regions.

**Figure 1:**
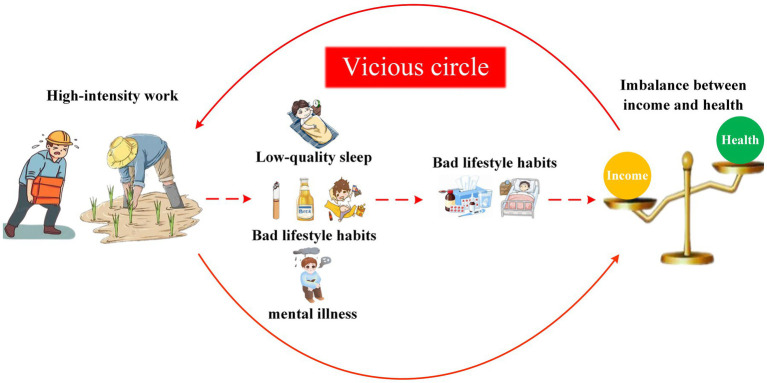
Mechanism of action diagram.

The mechanism of high-intensity work influencing the CCD among farmers is shown in Figure 1.

## 3 Data, variables, and method

### 3.1 Data source

This study utilized individual-level cross-sectional data from the 2018 China Labor Force Dynamics Survey (CLDS2018). The Center for Social Science Survey of Sun Yat-sen University was surveyed in 2018, employing a scientific sampling method involving multi-stage and multi-level probability sampling proportional to the size of the labor force. The data in this study encompassed 497 villages and dwellings across 29 provinces and cities in China, excluding Hong Kong, Macao, Taiwan, Tibet, and Hainan. This wide and representative survey coverage ensures the dataset’s richness and diversity. The primary focus of this research is to investigate the impact of high-intensity work on farmers’ incomes. Therefore, the study sample was limited to “rural residents aged 16 and above, engaged in work in the past year, and involved in agricultural or irregular work types (such as casual laborers, vendors, nannies without dispatching units, self-employed drivers, manual craftsmen, etc.).” After eliminating samples with significant missing data, 4,147 rural residents were included in the analysis using their questionnaire data. This study applied a logarithmic transformation to continuous variables and truncated them at the 1 and 99% percentiles to address potential issues with heteroskedasticity and the influence of extreme values.

### 3.2 Variables selection

#### 3.2.1 Explained variable

In this study, the primary explanatory variable is the CCD. Before calculating the CCD, it is necessary to construct composite indices for both health level and income level. Previous research on measuring health levels has primarily focused on self-assessed health, lifestyle habits, and the occurrence of diseases (19). Therefore, this study adopts a comprehensive approach by selecting six indicators from three dimensions encompassing individual physical and mental health, living habits, and injuries and diseases. These chosen indicators are used to construct a holistic health level index, and their specific details are presented in Table 1. The study of Kuang et al. (31) shows that the income level is jointly influenced by five aspects: human capital, natural capital, physical capital, financial capital, and social capital. So, this study selected ten indicators to construct a composite index of income level, and the specific indicators are shown in Table 2. Both composite indices were calculated using the entropy method.

**Table 1:** Health capital indicators for farmers.

Variable	Category	Index	Definition	Weight
Health capital	Physical and mental health	Subjective physical health status	Respondents’ self-rated health status (Very healthy = 5; healthy = 4; average = 3; relatively unhealthy = 2; very unhealthy = 1)	0.036
		Subjective mental health status	Total score of 20 mental health indicators added together^1^ (The higher the score, the worse the mental health level)	0.222
	Living habits	Smoking	Number of cigarettes smoked per day by the respondent	0.004
		Drinking	Frequency of alcohol consumption by respondents (never drink or have stopped drinking = 0; 1–2 times/week = 1; 3–4 times/week = 2; daily or almost daily = 3)	0.047
		Exercising	Number of exercises per week by the respondent	0.685
	Injuries	Attendance or hospitalization due to illness	What type of hospital the respondent visited or was hospitalized in the past 2 weeks due to illness (no visit = 0; village, township health facility = 1; community, district and county health facility = 2; prefecture-level city and above health facility = 3)	0.006

Mental health in CLDS2018 is assessed using the “Degree of Self-Depression” scale, which comprises 20 indicators. These indicators are as follows: “I am bothered by trivial matters,” “I experience a loss of appetite,” “Even with support from family and friends, I find it difficult to overcome the bitterness within,” “I feel inferior to most people,” “I experience feelings of depression,” “I struggle to concentrate on tasks,” “I feel despondent,” “I feel helpless,” “I perceive a lack of efficacy in my actions,” “I have a sense of hopelessness,” “I feel like a failure in life,” “I find it hard to escape from negative thoughts,” “I experience disrupted sleep patterns,” “I talk less than usual,” “I feel isolated and lonely,” “I believe that others treat me poorly,” “I perceive life as lacking purpose,” “I have moments of crying,” “I experience persistent worries,” “I feel disliked by others,” and “I struggle to maintain my daily routine.” Each of these 20 indicators is assigned a value ranging from 1 to 4, with higher values indicating a more severe mental health condition.

**Table 2:** Livelihood capital indicators for farmers (excluding health capital).

Variable	Category	Index	Definition	Weight
Livelihood capital	Human capital	Age	Age of Respondent	0.212
		Education	Th education level of respondent (Assign a value of 1–11, the higher the value, the higher the education level)	0.788
	Natural capital	Arable land area	Arable land area per capita in the respondent’s household	0.192
		Number of domestic animals	Number of domestic animals per capita in the respondent’s household	0.808
	Physical capital	Number of farm machinery	Number of farm machinery per capita in the respondent’s household	0.938
		Number of cars	Number of cars per capita in the respondent’s household	0.062
	Financial capital	Types of sources of income	Number of sources of household income of respondent	0.042
		Property income	Number of property income per capita in the respondent’s household	0.958
	Social capital	Social relations	The number of friends the respondent can get support and help	0.952
		Degree of familiarity	Respondents’ familiarity with their neighbors (very unfamiliar = 1; not very familiar = 2; fairly familiar = 3; more familiar = 4; very familiar = 5)	0.048

Mental health in CLDS2018 is assessed using the “Degree of Self-Depression” scale, which comprises 20 indicators. These indicators are as follows: “I am bothered by trivial matters,” “I experience a loss of appetite,” “Even with support from family and friends, I find it difficult to overcome the bitterness within,” “I feel inferior to most people,” “I experience feelings of depression,” “I struggle to concentrate on tasks,” “I feel despondent,” “I feel helpless,” “I perceive a lack of efficacy in my actions,” “I have a sense of hopelessness,” “I feel like a failure in life,” “I find it hard to escape from negative thoughts,” “I experience disrupted sleep patterns,” “I talk less than usual,” “I feel isolated and lonely,” “I believe that others treat me poorly,” “I perceive life as lacking purpose,” “I have moments of crying,” “I experience persistent worries,” “I feel disliked by others,” and “I struggle to maintain my daily routine.” Each of these 20 indicators is assigned a value ranging from 1 to 4, with higher values indicating a more severe mental health condition.

Drawing on Chen et al. (32), this study constructs a model of the CCD, as shown in Equations 1–3:


(1) $$C_{i}={\left\{\frac{{\text{Health}}_{i}*{\text{Income}}_{i}}{{\left[({\text{Health}}_{i}+{\text{Income}}_{i})/2\right]}^{2}}\right\}}^{\frac{1}{2}}$$

(2) $$T_{i}=\alpha *{\text{Health}}_{i}+\beta *{\text{Income}}_{i}$$

(3) $$D_{i}=\sqrt{C_{i}*T_{i}}$$

Where ${\text{Health}}_{i}$ and ${\text{Income}}_{i}$ represent the composite index of health level and composite index of income level of farmer i , respectively, calculated using the entropy method. $C_{i}$ , $T_{i}$ , and $D_{i}$ signify the degree of coupling, coordination, and coupling coordination between health and income of farmer i , respectively. The contribution coefficients α and β are assumed to be equal, reflecting the importance of health and income to farmers in this study. We consider the assessed subsystems to have an equal status; hence, α=β=1/2 .

Furthermore, in line with Chen et al. (32), farmers with a coupling coordination degree lower than 0.5 are classified as health-income imbalanced and assigned a value of 0; on the contrary, those with a degree higher than or equal to 0.5 are labeled as health-income balanced and assigned a value of 1.

#### 3.2.2 Core explanatory variable

The core explanatory variable in this study is the intensity of work, which is represented by whether the work is classified as high intensity (Work). Prior research has highlighted that the number of working hours is a significant component of work intensity (10, 33, 34). Hence, this study utilizes the weekly working hours to capture work intensity among farmers. As farmers’ weekly working hours are subject to variations, this study adopts the approach proposed by Lu et al. (35) and sets all farmers’ average weekly working hours as the threshold. If a farmer’s weekly working hours exceed this threshold, it is categorized as high-intensity work and assigned a value of 1. Conversely, if it falls below the threshold, it is classified as low-intensity work and assigned a value of 0.

#### 3.2.3 Control variables

Farmers’ health and income status are influenced not only by their circumstances but also by their family and village environment. Consequently, this study incorporates control variables from three dimensions: individual, household, and village. To prevent multicollinearity, variables already represented in the composite index of the health level and the composite index of income level are excluded as control variables in this study. In detail, at the individual level, control variables such as respondents’ gender, marital status, political affiliation, health insurance purchase, and internet usage were chosen. Total household population and labor force were considered control variables at the household level. Additionally, at the village level, control variables included the presence of non-agricultural industries, health rooms, squares or parks, and reforestation. Area dummies were also incorporated to account for environmental disparities across provinces.

The analysis of demographic control variables presents characteristics that accurately reflect contemporary rural China’s socioeconomic conditions. The gender distribution shows males accounting for 54.45% of the sample, slightly outnumbering females, corresponding with men’s typically more active role in agricultural production across many rural areas. The married population proportion reaches 91.87%, indicating the continued dominance of traditional marital values in rural communities. Communist Party members represent merely 3.67% of respondents, demonstrating the relatively low proportion of party membership in rural populations. Notably, only 11.89% of respondents reported having medical insurance, revealing substantial room for improvement in healthcare coverage expansion across rural regions. Internet usage is 44.35%, showing that while digital access has grown in rural areas, a significant gap remains compared to urban centers. Family size statistics show that 67.27% of households have five members or fewer, while 93.32% report five or fewer working-age family members, confirming that small-scale family farming remains the predominant household structure, consistent with China’s fundamental agricultural profile. These demographic control variable distributions collectively and comprehensively represent the actual conditions of rural China’s population structure, economic industries, infrastructure development, and resident living standards.

#### 3.2.4 Instrumental variable

The peer effect suggests that interactions within a group have an impact on the behavior of individuals within the group (36), and this influence of peer behavior on individual behavior is more common in rural areas (37, 38). Therefore, this study concludes that whether farmers work at high intensity or not is influenced by the intensity of work of farmers in the same village. Referring to Xiao et al. (39), this study defines the instrumental variable of whether respondents work at high intensity or not as taking the weight of the number of people who work at high intensity in their villages and the respondents themselves. The formula is as Equation 4:


(4) $$\mathrm{IV}=\frac{N}{T-1}$$

Where *IV* is the instrumental variable, N denotes the number of people representing high-intensity work in the same village, and T represents the total number of people there. The definitions of the variables are shown in Table 3.

**Table 3:** Variable meaning and descriptive statistics.

Variable	Definition	Mean	S. D.
Work	Whether the respondent works intensely (Yes = 1; No = 0)	0.504	0.500
CCD	Whether the respondent’s health-income coupled and coordinated (Yes = 1; No = 0)	0.143	0.351
Gender	Gender of respondents (Male = 1; Female = 0)	0.544	0.498
Marriage	Whether the respondent is married (Yes = 1; No = 0)	0.919	0.273
Party	Whether the respondent is a member of the Chinese Communist Party (Yes = 1; No = 0)	0.037	0.188
Insurance	Whether the respondent has health insurance (Yes = 1; No = 0)	0.119	0.324
Internet	Whether respondent uses the Internet (Yes = 1; No = 0)	0.443	0.497
Pop_total	Total number of respondents’ households	3.429	2.052
Pop_labor	Total labor force in respondents’ households	1.140	1.446
Off-farm	Whether the respondent’s village has non-agricultural industries (Yes = 1; No = 0)	0.174	0.379
Sanitary	Whether the respondent’s village has a health center/institution (Yes = 1; No = 0)	0.831	0.375
Park	Whether the respondent’s village has a plaza or park (Yes = 1; No = 0)	0.468	0.499
Land	Whether the respondent’s village carries out the return of farmland to forests (Yes = 1; No = 0)	0.577	0.494

### 3.3 Model setting

The main objective of this study is to examine the impact of high-intensity work on the CCD among farmers. However, whether farmers engage in intense work can be influenced by factors such as gender and family size and unobservable factors such as laziness and stress tolerance. This self-selection process introduces non-random characteristics, leading to potential bias issues and inconsistent estimation results if not adequately addressed. To tackle this problem, many researchers have turned to propensity score matching (PSM) as a solution (40, 41). Nevertheless, PSM has limitations, as it only considers selective bias caused by observable factors and overlooks the influence of unobservable factors (42).

This study adopts the approach used by Ma et al. (43) and incorporates the endogenous transformed probit model (ESP) to address the selectivity bias stemming from both observable and unobservable factors. This empirical method allows us to examine the impact of high-intensity work on the CCD among farmers with more robustness and accuracy. The ESP model consists of two stages. In the first stage, we estimate the probability of farmers opting for high-intensity work using the Probit model. We assume that farmers are risk-neutral and will choose high-intensity work if its utility surpasses the utility of low-intensity work. The specific formula is as follows:


(5) $$I_{i}^{*}={\mathrm{\gamma Z}}_{i}+u_{i},I_{i}=\{\begin{array}{c} 1,\text{if}\;I_{i}^{*}>0\;\\ \;0,\text{otherwise} \end{array}\;$$

Equation 5 represents the choice equation. Here, $I_{i}^{*}$ denotes the probability that a farmer chooses high-intensity work, determined by $I_{i}$ . For farmers who work at high intensity, $I_{i}$ takes the value of 1; conversely, it takes the value of 0. $Z_{i}$ is a set of variables that influence the intensity of farmers’ work. Parameter γ is to be estimated, indicating the effect of each variable on the intensity of farmers’ work. The term $u_{i}$ represents the random error term.

The second stage of the ESP model is to estimate the impact of high-intensity work on the CCD among farmers. The specific equations are as follows:


(6) $$I_{1i}^{*}=\beta_{1i}X_{1i}+\varepsilon_{1i},Y_{1i}=\{\begin{array}{c} 1,\text{if}\;I_{1i}^{*}>0\;\\ \;0,\text{otherwise} \end{array}\;\text{for}\;I_{i}=1$$

(7) $$I_{0i}^{*}=\beta_{0i}X_{0i}+\varepsilon_{0i},Y_{0i}=\{\begin{array}{c} 1,\text{if}\;I_{0i}^{*}>0\;\\ \;0,\text{otherwise} \end{array}\;\text{for}\;I_{i}=0$$

Equations 6, 7 represent the outcome equations. $I_{1i}^{*}$ and $I_{0i}^{*}$ represent the probability of health-income balance for farmers with high-intensity and low-intensity work, respectively. $X_{1i}$ and $X_{0i}$ capture the factors influencing the health-income balance for farmers with high-intensity and low-intensity work, respectively. $Y_{1i}$ and $Y_{0i}$ indicate whether the health income of farmers with high-intensity work and low-intensity work is balanced, which can be obtained from the sample data. If it is balanced, they are assigned a value of 1; vice versa, they are assigned a value of 0. $\beta_{1i}$ and $\beta_{0i}$ are the parameters to be estimated. $\varepsilon_{1i}$ and $\varepsilon_{0i}$ are random error terms.

The ESP model requires that the instrumental variables included in $Z_{i}$ during the first-stage estimation must not be accounted for by X_1i_ or X_0i_ in the second stage (44). As previously mentioned, this study selects the weight of the people working at high intensity in the respondent’s village, other than the respondent himself, as an instrumental variable to address the endogeneity related to farmers’ high-intensity work. After estimating the correlation coefficients using the ESP model, it becomes possible to calculate three average treatment effects of high-intensity work on the CCD: the average treatment effect for the treatment group (ATT), the control group (ATU), and the overall sample (ATE). However, this study primarily examines how farmers’ CCD changes after engaging in high-intensity work and whether it leads to a vicious cycle. As a result, the estimation results of ATU and ATE are of limited relevance to this study. Therefore, the study exclusively estimated ATT to measure the impact of high-intensity work on the CCD among farmers.

## 4 Empirical results and discussion

### 4.1 The mean difference between farmers with high and low-intensity work

In this study, the disparities between farmers with high-intensity work and low-intensity work were analyzed using a t-test of difference in means, and the results are presented in Table 4. The findings reveal that farmers with high-intensity work exhibit a lower degree of CCD than those with low-intensity work. The differences in the means of the control and instrumental variables were statistically significant at the 10% level, except for the variables Marriage, Party, Off-farm, and Park. These results indicate a substantial distinction between farmers who engaged in high-intensity work and those who opted for low-intensity work. Nevertheless, as the t-test for difference in means cannot provide insights into whether these discrepancies are attributed to high-intensity work, we undertook a rigorous empirical investigation utilizing the more scientifically sound ESP model. We aimed to delve into the impact of high-intensity work on the CCD among farmers while considering the potential bias introduced by self-selection in the study sample.

**Table 4:** Mean difference of each variable.

Variable	Not high-intensity work	High-intensity work	Diff.
CCD	0.159	0.129	0.030^***^
Gender	0.512	0.576	−0.064^***^
Marriage	0.917	0.920	−0.003
Party	0.035	0.039	−0.004
Insurance	0.109	0.129	−0.020^*^
Internet	0.418	0.468	−0.050^***^
Lnpop_total	1.459	1.496	−0.037^**^
Lnpop_labor	1.119	1.161	−0.042^***^
Off-farm	0.173	0.175	−0.002
Sanitary	0.861	0.802	0.059^***^
Park	0.472	0.465	0.007
Land	0.406	0.661	−0.255^***^
IV	0.404	0.663	−0.259^***^

***, ** and * represent the significance levels of 1, 5 and 10%, respectively.

### 4.2 Estimation results of the ESP model of factors influencing work intensity

The “Selection” column results in Table 5 indicate that male farmers are more likely to choose high-intensity work than female farmers. This gender difference can be attributed to rural China’s traditional division of labor, where women predominantly take on household responsibilities. At the same time, men are primarily responsible for earning income to support the family outside the home (45). Moreover, the analysis reveals that farmers with medical insurance are more inclined to opt for high-intensity work. This observation is likely because health insurance helps mitigate potential income losses resulting from health issues (46), thereby reducing the risk of experiencing a decline in income due to engaging in high-intensity work for farmers.

**Table 5:** ESP model estimation results of influencing factors of work intensity and CCD.

Variable	Selection	CCD	
		High-intensity work	Not high-intensity work
Gender	0.154^***^ (0.043)	−0.039 (0.075)	0.048 (0.073)
Marriage	0.063 (0.082)	−0.067 (0.136)	−0.080 (0.130)
Party	−0.037 (0.111)	0.358^**^ (0.159)	0.754^***^ (0.169)
Insurance	0.114^*^ (0.068)	0.181^*^ (0.101)	0.389^***^ (0.104)
Internet	0.051 (0.044)	0.189^**^ (0.073)	0.216^***^ (0.072)
Lnpop_total	0.054 (0.075)	−0.240^**^ (0.122)	0.050 (0.119)
Lnpop_labor	0.013 (0.072)	0.150 (0.123)	−0.222^**^ (0.111)
Off-farm	−0.058 (0.065)	0.218^*^ (0.112)	−0.043 (0.108)
Sanitary	−0.022 (0.066)	−0.147 (0.096)	−0.148 (0.114)
Park	−0.022 (0.51)	0.049 (0.082)	0.166^**^ (0.078)
Land	0.032 (0.052)	0.104 (0.087)	−0.426^***^ (0.085)
IV	2.590^***^ (0.107)	–	–
Area dummies	Yes	Yes	Yes
_cons	−1.630^***^ (0.186)	−0.957^***^ (0.286)	−0.364 (0.274)
rho1	0. 255^**^ (0.122)		
rho0	0. 086 (0.103)		
Wald test of indep. eqns.	4.680^*^		
Log pseudolikelihood	−3903.030		
Observation	4,147		

***, ** and * represent the significance levels of 1, 5 and 10%, respectively.

The regression coefficient for the instrumental variable (IV) at the bottom of the “Selection” column is significantly positive, indicating that the work situation of other farmers in the same village significantly influences whether a farmer chooses to work at high intensity. However, when the Probit model was used to estimate the effect of the instrumental variable on the CCD, the estimate was not statistically significant (coefficient of −0.137, *p*-value of 0.195). To further examine the role of high-intensity work on the CCD among farmers, the instrumental variable model (IV-2SLS) was employed. The first-stage *F*-value of 34.34 suggests that the instrumental variable used was not weak, supporting its appropriateness in the analysis. In conclusion, the instrumental variables selected for this study were deemed appropriate, as they exhibited significant influence on farmers’ choice of high-intensity work and were robust in the instrumental variable model estimation.

### 4.3 Estimation results of ESP model of factors influencing the CCD

The “High-intensity work” and “Not high-intensity work” columns of Table 5 present the factors influencing the CCD for farmers engaged in high-intensity and low-intensity work, respectively. The results show that among the control variables, “Party,” “Insurance,” and “Internet” significantly contribute to the CCD of both high-intensity work and low-intensity work farmers, which aligns with the findings of Morduch and Sicular (47) that Party-affiliated farmers tend to have higher average incomes, facilitating health-income balance. As mentioned earlier, health insurance plays a role in reducing income losses caused by health issues (46), thereby improving the CCD. The Internet can enhance farmers’ income opportunities (48) and enrich their lives, providing access to entertainment and promoting mental health, supporting the development of health-income balance. Total household size and total household labor force size have a negative impact on the CCD of high-intensity and low-intensity working farmers, respectively. For high-intensity working farmers, a larger family size implies a heavier responsibility to earn money and support the family, increasing the likelihood of facing the challenge of imbalanced health and rising income. On the other hand, for farmers with low-intensity work, a larger household labor force means a reduced burden of earning income to support the family, potentially leading to an imbalance of improved health but declining income. Furthermore, the variables “Off-farm” and “Park” among the control variables positively influence the CCD of high-intensity and low-intensity farmers. A well-developed non-farm industry in villages boosts farmers’ income and reduces the likelihood of them experiencing occupational diseases during agricultural production (49). The presence of parks and squares in the villages provides farmers with free opportunities to engage in physical exercise, thereby contributing to their overall physical health. On the other hand, whether villages undertake reforestation work negatively impacts the CCD of low-intensity working farmers. Villages engaging in reforestation initiatives may encourage farmers to seek alternative job opportunities outside the agricultural sector (50). This increase in external employment might escalate farmers’ work pressure and cost of living (51, 52), resulting in a potentially vicious cycle of imbalanced health and income.

The Wald test values for the independence of the equations, as shown below in Table 5, are statistically significant at the 10% level, leading to the rejection of the initial hypothesis that the selection and outcome equations are independent. The estimated value of rho1 is also statistically significant at the 5% level, indicating the presence of unobservable factors that simultaneously influence both farmers’ decision to work intensely and the degree of their CCD, which suggests that the baseline regression model may be affected by self-selection bias. The utilization of an endogenous switching model is appropriate in this context.

### 4.4 Estimation results of the mean treatment effect of high-intensity work on the CCD

Table 6 presents the average treatment estimate (ATT) of the impact of high-intensity work on farmers’ CCD. The ATT is calculated to be −0.011 and is statistically significant at the 1% level, which means that for farmers who are currently working at high intensity when they change from high-intensity to low-intensity work, the CCD harmonization goes up by 0.011 and relatively by 8.527%. Therefore, high-intensity work negatively affects farmers’ CCD and may lead them into a cycle of health-income imbalance, thus confirming hypothesis H1.

**Table 6:** Estimated results of ATT.

Variable	High-intensity work	Not high-intensity work	ATT	Change (%)
High-intensity work	0.129	0.140	−0.011^***^ (0.002)	8.527

*** represent the significance levels of 1%.

### 4.5 Robustness test

#### 4.5.1 Exclusion of samples

As farmers age, they may experience a decline in physical functioning due to age-related factors (25). Including older farmers in the sample could lead to biased estimates of the average treatment effect (ATT), as calculated in the previous section. A study in rural China found that farmers over 70 are more likely to suffer from old age diseases (53). The present research reevaluated the ATT of high-intensity work on farmers’ CCD by excluding the sample of farmers over 70 years of age to address this potential bias. The results are presented in Table 7. It can be observed that even after excluding the sample of farmers over 70 years of age, high-intensity work continues to impact farmers’ CCD negatively. Specifically, for farmers currently working at high intensity, the CCD rises by 0.025 and relatively by 19.531% when they change from high-intensity to low-intensity work, which indicates that the findings of this paper are robust.

**Table 7:** Robustness analysis results.

Method	High-intensity work	Not high-intensity work	ATT/ATU	Change (%)
Delete samples over 70 years old	0.128	0.153	−0.025^***^ (0.002)	19.531

*** represent the significance levels of 1%.

#### 4.5.2 PSM estimates

To rigorously verify the robustness of our findings, we employ propensity score matching (PSM) to estimate the impact of work intensity on the CCD. Table 8 presents the PSM results using 1:1 nearest-neighbor matching. The estimates remain consistent with our baseline regression in both direction and significance. The PSM results indicate that reducing work intensity from high to low levels would increase farmers’ CCD by 0.045 points, representing a 35.156% relative improvement. This robust evidence confirms the stability of our core findings across different estimation methods.

**Table 8:** The results of PSM.

Method	High-intensity work	Not high-intensity work	ATT	Change (%)
PSM	0.128	0.173	−0.045^***^ (0.014)	35.156

*** represent the significance levels of 1%.

### 4.6 Heterogeneity results of high-intensity work influencing the CCD

#### 4.6.1 Labor mobility heterogeneity

Farmers who work close to their homes tend to have a more familiar lifestyle and culture and greater social and family support, reducing work stress and higher life satisfaction. Therefore, this study hypothesizes that the impact of high-intensity work on the CCD varies among farmers in different workplaces. To investigate this heterogeneity, the study groups farmers based on whether they work in their village. As presented in Table 9, the results show that for farmers who work in their village, when they switch from high to low-intensity work, the CCD rises by 0.018 and relatively by 14.39%. Conversely, for farmers who do not work in the village, the CCD rises by 0.036 and relatively by 25.2% when they change from high-intensity work to low-intensity work. These findings indicate that the inhibitory effect of high-intensity work on the CCD is more substantial for farmers who work outside the village, thus validating hypothesis H2a.

**Table 9:** Results of the analysis of labor mobility heterogeneity.

Classification		ATT	Chang (%)
Place of work	Native community	−0.018^***^ (0.002)	14.393
	Non-native community	−0.036^**^ (0.018)	25.200

*** and **represent the significance levels of 1 and 5%, respectively.

#### 4.6.2 Labor market heterogeneity

To further examine labor market heterogeneity in the effects of high-intensity work on the CCD, we conduct subsample analyses comparing eastern and non-eastern regions. As presented in Table 10, the results show that when eastern farmers switch from high to low-intensity work, the CCD reduces by 0.035 and relatively by 22.152%. Conversely, when non-eastern farmers change from high-intensity work to low-intensity work, the CCD rises by 0.024 and relatively by 20.339%. These opposing effects confirm the more substantial inhibitory impact of high-intensity work on the CCD in developed eastern regions and provide conclusive evidence validating hypothesis H2b regarding labor market segmentation.

**Table 10:** Results of the analysis of labor market heterogeneity.

Classification		ATT	Chang (%)
Region	Eastern region	0.035^***^ (0.005)	22.152
	Non-Eastern region	−0.024^***^ (0.002)	20.339

*** represent the significance levels of 1%.

## 5 Conclusions and recommendation

This study aims to investigate the determinants of farmers’ high-intensity work, analyze the impact of high-intensity work on the CCD, and examine the heterogeneity of this impact among different groups of farmers. The individual cross-sectional data utilized for empirical analysis were obtained from the 2018 China Labor Dynamics Survey (CLDS2018). This study used the endogenous switching probit model (ESP) as the empirical model to address potential bias arising from sample self-selection. The study reveals three key findings about work intensity and the CCD. First, farmers engaged in high-intensity labor show significantly lower CCD scores (mean = 0.129) than low-intensity workers (mean = 0.159), with a notable 0.030-point gap. Second, reducing work intensity from high to low levels increases CCD by 8.527%, demonstrating strenuous labor’s suppressive effect on the CCD. Third, this negative effect is more substantial for farmers who work off-site and in non-Eastern regions. These findings expose fundamental tensions in China’s rural development. The urban–rural divide forces farmers to compensate for low productivity through extended work hours, as they lack sufficient human and social capital. Simultaneously, inadequate rural social security systems fail to protect farmers from health risks, causing medical expenses to erode income gains. Migrant workers bear particularly severe consequences, which stem from three intersecting vulnerabilities: labor market discrimination that suppresses wages, welfare restrictions tied to household registration, and psychological strain from severed community ties. This isolation deprives them of emotional support systems in urban environments, worsening health outcomes and work performance while deepening health-income imbalances.

This study’s findings about the complex relationship between high-intensity work and the CCD reflect fundamental challenges in rural China’s development and demand comprehensive policy solutions. The government should implement coordinated interventions across three key areas. First, the urban–rural divide limits farmers’ human and social capital, which forces them to rely on strenuous labor for income growth. Policymakers must prioritize rural education and vocational training. Local governments should develop technical training programs tailored to regional industries, enhancing productivity and reducing dependence on intensive labor. Second, China must strengthen rural social security systems. Inadequate health coverage makes medical expenses undermine income-health balance. The central government should increase rural medical infrastructure investments, improving primary care quality. Simultaneously, authorities must streamline insurance reimbursement procedures and raise coverage rates, protecting farmers from medical impoverishment. Third, migrant workers face wage discrimination, welfare exclusion, and psychological stress. Labor regulators must enforce equal pay policies and safeguard workers’ rights. The healthcare system should achieve seamless cross-regional insurance portability, which ensures migrant workers’ access to medical services. Community organizations and NGOs can establish support platforms that provide counseling and facilitate urban integration to alleviate mental health pressures.

With China’s successful completion of the poverty eradication task in 2020 and the commencement of the era of comprehensive rural revitalization, increasing farmers’ income and overall prosperity has become a significant objective in the country’s rural development efforts. However, farmers face challenges in finding avenues to boost their income, which are constrained by social capital and education level. As a result, they often resort to intensifying their work on existing income-generating channels as a means to achieve sustainable income growth. However, this approach also exposes them to substantial risks, particularly the risk of falling into poverty due to health issues. In light of these challenges, drawing on unique survey data and employing empirical modeling, this research examines both the positive and negative impacts of high-intensity work on farmers’ wealth. It offers fresh insights into how farmers rely on increasing work intensity to pursue wealth while shedding light on this approach’s potential risks and implications. However, several areas warrant further research in the future: (1) Our study faces data limitations that restrict work intensity measurement to working hours alone. This operationalization presents conceptual constraints, as work intensity combines multiple dimensions, including task complexity, environmental conditions, and psychological stress. For instance, some jobs require short but cognitively demanding tasks, which may constitute high-intensity labor despite brief durations. Future research should develop composite intensity metrics integrating working hours, job content characteristics, workplace environments, and stress indicators. Such multidimensional measures yield more accurate assessments that better reflect real-world conditions, which can ultimately strengthen policy recommendations. (2) Future research could shift the focus to migrant workers to gain deeper insights. Migrant workers experience greater work and life pressures compared to farmers engaged in agricultural activities. They are more susceptible to “not seeking treatment for minor illnesses but unable to afford major ones.” Additionally, migrant workers play a crucial role in China’s social stability and urban–rural integration, making them a group worthy of special attention. (3) The empirical analyses in this paper were limited by the data, which lacked standardized health indicators, such as the PSQI Sleep Quality Index or the GHQ-12 Mental Health Score, and thus did not allow for mediation analyses. Future studies could greatly advance this research by using a longitudinal design that collects biomarker data, validated psychosocial scales, and geospatial healthcare accessibility measures.

## Data Availability

The data analyzed in this study is subject to the following licenses/restrictions: the datasets used and/or analyzed during the current study are available from the corresponding author upon reasonable request. Requests to access these datasets should be directed to 2332350003@ynnu.edu.cn.

## Footnotes

^1^On February 25, 2021, Chinese leaders announced a resounding success in China’s battle against poverty, achieving comprehensive victory: http://www.gov.cn/xinwen/2021-02/25/content_5588866.htm#1

^2^“Strategic Plan for Rural Revitalization (2018–2022)”: http://www.gov.cn/zhengce/2018-09/26/content_5325534.htm
